# Practices and activities among healthcare personnel with severe acute respiratory coronavirus virus 2 (SARS-CoV-2) infection working in different healthcare settings—ten Emerging Infections Program sites, April–November 2020

**DOI:** 10.1017/ice.2021.262

**Published:** 2022-08

**Authors:** Nora Chea, Taniece Eure, Austin R. Penna, Cedric J. Brown, Joelle Nadle, Deborah Godine, Linda Frank, Christopher A. Czaja, Helen Johnston, Devra Barter, Betsy Feighner Miller, Katie Angell, Kristen Marshall, James Meek, Monica Brackney, Stacy Carswell, Stepy Thomas, Lucy E. Wilson, Rebecca Perlmutter, Kaytlynn Marceaux-Galli, Ashley Fell, Sarah Lim, Ruth Lynfield, Sarah Shrum Davis, Erin C. Phipps, Marla Sievers, Ghinwa Dumyati, Cathleen Concannon, Kathryn McCullough, Amy Woods, Sandhya Seshadri, Christopher Myers, Rebecca Pierce, Valerie L. S. Ocampo, Judith A. Guzman-Cottrill, Gabriela Escutia, Monika Samper, Sandra A. Pena, Cullen Adre, Matthew Groenewold, Nicola D. Thompson, Shelley S. Magill

**Affiliations:** 1 Division of Healthcare Quality Promotion, National Center for Emerging and Zoonotic Infectious Diseases, Centers for Disease Control and Prevention, Atlanta, Georgia; 2 Chenega Professional & Technical Services, Chesapeake, Virginia; 3 California Emerging Infections Program, Oakland, California; 4 Colorado Department of Public Health and Environment, Denver, Colorado; 5 Epidemic Intelligence Service, Centers for Disease Control and Prevention, Atlanta, Georgia; 6 Connecticut Emerging Infections Program, Yale School of Public Health, New Haven, Connecticut; 7 Georgia Emerging Infections Program, Atlanta Veterans Affairs Medical Center, Foundation for Atlanta Veterans Education and Research, Atlanta, Georgia; 8 Georgia Emerging Infections Program, Emory University School of Medicine, Atlanta, Georgia; 9 Maryland Department of Health, Baltimore, Maryland; 10 Minnesota Department of Health, St Paul, Minnesota; 11 New Mexico Emerging Infections Program, University of New Mexico, Albuquerque, New Mexico; 12 New Mexico Department of Health, Santa Fe, New Mexico; 13 New York Emerging Infections Program, University of Rochester Medical Center, Rochester, New York; 14 Public Health Division, Oregon Health Authority, Portland, Oregon; 15 Tennessee Department of Health, Nashville, Tennessee; 16 Division of Field Studies and Engineering, National Institute for Occupational Safety and Health, Centers for Disease Control and Prevention, Cincinnati, Ohio

## Abstract

Healthcare personnel with severe acute respiratory coronavirus virus 2 (SARS-CoV-2) infection were interviewed to describe activities and practices in and outside the workplace. Among 2,625 healthcare personnel, workplace-related factors that may increase infection risk were more common among nursing-home personnel than hospital personnel, whereas selected factors outside the workplace were more common among hospital personnel.

Healthcare personnel (HCP) are at risk for severe acute respiratory coronavirus virus 2 (SARS-CoV-2) infection because of close contact with persons with coronavirus disease 2019 (COVID-19) in and outside the workplace. Given the diversity of the HCP workforce and the settings in which they live and work, the risk for acquisition of SARS-CoV-2 infection may vary. We analyzed data from HCP with SARS-CoV-2 infection included in surveillance conducted by the Centers for Disease Control and Prevention (CDC) Emerging Infections Program (EIP).^1^ We compared characteristics of HCP cases working in hospitals (H-HCP), nursing homes (NH-HCP) or other healthcare facilities to identify differences in factors potentially affecting SARS-CoV-2 infection risk.

## Methods

In April 2020, to conduct surveillance for SARS-CoV-2 infections in HCP, the CDC began collaborating with 10 EIP sites: California, Colorado, Connecticut, Georgia, Maryland, Minnesota, New Mexico, New York, Oregon, Tennessee. Among them, 7 sites conducted sentinel surveillance in selected hospitals or nursing homes: 5 sites in hospitals only and 2 sites in hospitals and nursing homes. Two sites conducted population-based surveillance among HCP who resided in selected counties, and the remaining site conducted sentinel surveillance in selected hospitals and population-based surveillance among NH-HCP in a single county (Table 1).

**Table 1.** Characteristics of Healthcare Personnel (HCP) With SARS-CoV-2 Infection by Primary Work Setting, April–November 2020

Characteristic	Hospital (N = 1,225)	Nursing Home (N = 604)	Other Facility^a^ (N = 789)	All Facilities (N = 2,625)^b^
Age, median y (IQR)^c^	38 (29–49)	42 (31–53)	40 (29–51)	39 (29–51)
**Sex, no. (%)**				
Female	931 (76.0)	505 (83.6)	650 (82.4)	2,090 (79.6)
Male	286 (23.3)	95 (15.7)	136 (17.2)	517 (19.7)
Other or not reported	8 (0.7)	4 (0.7)	3 (0.4)	18 (0.7)
**Site, no. (%)**^d^				
California	221 (18.0)	244 (40.4)	407 (51.6)	873 (33.3)
Colorado	122 (10.0)	16 (2.6)	53 (6.7)	191 (7.3)
Connecticut	63 (5.1)	1 (0.2)	5 (0.6)	69 (2.6)
Georgia	251 (20.5)	87 (14.4)	235 (29.8)	578 (22.0)
Maryland	84 (6.9)	0	8 (1.0)	92 (3.5)
Minnesota	76 (6.2)	9 (1.5)	17 (2.2)	103 (3.9)
New Mexico	71 (5.8)	0	24 (3.0)	95 (3.6)
New York	156 (12.7)	214 (35.4)	22 (2.8)	392 (14.9)
Oregon	153 (12.5)	0	10 (1.3)	163 (6.2)
Tennessee	28 (2.3)	33 (5.5)	8 (1.0)	69 (2.6)
**Race and ethnicity, no. (%)**				
White, non-Hispanic	575 (46.9)	169 (28.0)	202 (25.6)	949 (36.2)
Hispanic or Latino, any race or races	227 (18.5)	128 (21.2)	314 (39.8)	670 (25.5)
Black, non-Hispanic	235 (19.2)	177 (29.3)	145 (18.4)	557 (21.2)
Asian, non-Hispanic	106 (8.7)	96 (15.9)	75 (9.5)	277 (10.6)
Other or multiple races, non-Hispanic	18 (1.5)	13 (2.2)	15 (1.9)	46 (1.8)
Native Hawaiian/Pacific Islander, non-Hispanic	15 (1.2)	6 (1.0)	12 (1.5)	33 (1.3)
American Indian/Alaska Native, non-Hispanic	12 (1.0)	0	1 (0.1)	13 (0.5)
Race or ethnicity not reported	37 (3.0)	15 (2.5)	25 (3.2)	80 (3.0)
**Healthcare role, no. (%)** ^e^				
Registered nurse	426 (34.8)	53 (8.8)	71 (9.0)	550 (21.0)
Nursing assistant or patient care technician	113 (9.2)	235 (38.9)	38 (4.8)	386 (14.7)
Administrative personnel	130 (10.6)	47 (7.8)	137 (17.4)	314 (12.0)
Home health aide or caregiver	2 (0.2)	30 (5.0)	131 (16.6)	163 (6.2)
Medical assistant	23 (1.9)	4 (0.7)	117 (14.8)	144 (5.5)
Licensed practical or vocational nurse	11 (0.9)	75 (12.4)	22 (2.8)	108 (4.1)
Physician	76 (6.2)	2 (0.3)	24 (3.0)	103 (3.9)
Food services staff	36 (2.9)	31 (5.1)	4 (0.5)	71 (2.7)
Environmental services staff	41 (3.3)	25 (4.1)	4 (0.5)	70 (2.7)
Facilities staff	41 (3.3)	21 (3.5)	7 (0.9)	69 (2.6)
Surgical or medical technician	32 (2.6)	6 (1.0)	21 (2.7)	59 (2.2)
Physical therapist or assistant	26 (2.1)	14 (2.3)	5 (0.6)	45 (1.7)
Dental practitioner or dental clinic worker	2 (0.2)	0	42 (5.3)	44 (1.7)
Pharmacy personnel	22 (1.8)	1 (0.2)	19 (2.4)	42 (1.6)
Nurse practitioner	22 (1.8)	6 (1.0)	12 (1.5)	40 (1.5)
Other	221 (18.0)	54 (8.9)	134 (17.0)	409 (15.6)
Not reported	1 (0.1)	0	1 (0.1)	8 (0.3)
**Underlying conditions, no. (%)**				
At least one underlying condition^f^	817 (66.7)	437 (72.4)	581 (73.6)	1,836 (69.9)
Obesity or severe obesity	375 (30.6)	236 (39.1)	292 (37.0)	903 (34.4)
Hypertension	151 (12.3)	118 (19.5)	147 (18.6)	416 (15.8)
Asthma	159 (13.0)	71 (11.8)	113 (14.3)	343 (13.1)
Diabetes mellitus	60 (4.9)	56 (9.3)	63 (8.0)	179 (6.8)
Current or recent smoker^g^	55 (4.5)	71 (11.8)	51 (6.5)	177 (6.7)
Autoimmune or rheumatologic disease	54 (4.4)	21 (3.5)	29 (3.7)	104 (4.0)
Heart condition	35 (2.9)	21 (3.5)	20 (2.5)	76 (2.9)
Pregnancy	31 (2.5)	7 (1.2)	23 (2.9)	61 (2.3)
Chronic obstructive pulmonary disease	7 (0.6)	7 (1.2)	2 (0.3)	16 (0.6)
Chronic kidney disease	2 (0.2)	2 (0.3)	3 (0.4)	7 (0.3)

Note. IQR, interquartile range.

a Includes HCP working in home health, outpatient facilities, assisted living facilities, mental health facilities (including psychiatric hospitals), and other nonhospital, non–nursing-home healthcare settings.

b 7 healthcare personnel did not report the type of facility in which they worked but did report other data included in the table.

c Excludes 27 HCP for whom age was not reported (13 working in hospitals, 6 in nursing homes, 4 in other facilities, and 4 with missing facility information).

d California and Georgia EIPs conducted surveillance in HCP residing in 3 San Francisco-area counties and 5 metropolitan Atlanta counties, respectively. Colorado, Connecticut, Maryland, Minnesota, New Mexico, New York, Oregon and Tennessee EIPs each recruited a convenience sample of hospitals; Colorado and Tennessee EIP also recruited convenience samples of nursing homes. In addition to recruiting a convenience sample of hospitals, New York EIP conducted surveillance among HCP working in nursing homes and residing in Monroe County. Cases reported by participating healthcare facilities could have worked in other settings affiliated with the facility, such as nursing homes or outpatient clinics. Cases were grouped based on the primary facility in which they reported working.

e 129 HCP reported multiple roles; these were resolved to a single role by reviewing available data (eg, descriptions of “other” roles), considering the amount of direct or indirect patient contact expected for a given role. Generally, in the absence of more information, the role expected to involve more patient contact was assigned.

f In addition to the conditions listed, includes active cancer, other immunosuppressive conditions, liver disease, other lung disease, and other reported conditions.

g Recent smokers were defined as HCP who quit smoking <1 year before the interview date.

Healthcare personnel cases were defined as persons serving in healthcare settings with the potential for direct or indirect exposures to patients or infectious materials^2^ and with a positive SARS-CoV-2 test on or after April 1, 2020. Health departments or facilities provided lists of HCP tested for SARS-CoV-2 from which cases were identified. EIP staff administered a questionnaire to English- or Spanish-speaking HCP who agreed to participate, usually by telephone using paper or electronic forms, to collect demographics and selected occupational and non–work-related activities and practices in the 14 days before COVID-19 symptom onset or, for asymptomatic HCP, the date of specimen collection for the positive test.

Data collection included information about whether HCP had close contact with persons with COVID-19. We made updates to the definition of close contact as necessary to align with current CDC guidance, which evolved over time. The timing of implementing these updates varied among EIP sites and depended on factors such as institutional review board approval. The process used by EIP site interviewers to communicate the definition of close contact to HCP also varied; some provided the definition to the HCP before asking questions pertaining to close contact, and others provided the definition only if the HCP requested clarification.

Data were entered into a CDC-developed REDCap database. We analyzed data available by November 16, 2020, grouping HCP based on their primary facility (ie, hospital, nursing home, or other facility type, such as outpatient clinics or home health). Characteristics of H-HCP and NH-HCP were compared using mid-*P* exact tests with statistical significance defined as *P* ≤ .05. Analyses were conducted using SAS version 9.4 software (SAS Institute, Cary, NC) and OpenEpi.^3^

This activity was reviewed by CDC and was conducted consistent with applicable federal law and CDC policy (45 C.F.R. part 46.102(l)(2), 21 C.F.R. part 56; 42 U.S.C. §241(d); 5 U.S.C. §552a; 44 U.S.C. §3501 et seq). EIP sites and participating facilities either deemed the project to be a nonresearch activity or obtained institutional review board approval.

## Results

### Healthcare personnel participation

As of November 13, 2020, a total of 8,175 cases had been reported to EIP sites: 2,773 were interviewed and 5,402 declined to participate, were not eligible (eg, did not meet the HCP definition), could not be reached, had not yet been contacted for interview, or spoke a language other than English or Spanish. Data for 2,637 of the interviewed HCP were available and were exported on November 16, 2020. However, 12 HCP were excluded: 8 with positive serology tests only, 3 with missing viral test collection dates, and 1 with a viral test collection date before April 2020. Among 2,625 cases in the analysis, the median time from positive SARS-CoV-2 viral test collection date to interview was 19 days (interquartile range [IQR], 9–35 days).

Of the 2,625 cases, 1,225 H-HCP (46.7%) were from 127 hospitals, 604 NH-HCP (23.0%) were from 145 nursing homes, and 789 HCP (30.1%) were from 249 other facilities (Table 1). Also, 7 HCP (0.3%) did not report their facility type. Most cases (70%) were reported from California, Georgia, or New York.

### Demographic characteristics, comorbidities, and healthcare roles

NH-HCP were more likely than H-HCP to report their race and ethnicity as either non-Hispanic Black or Asian, or Hispanic or Latino (66.4% vs 46.4%; *P* < .001) and to report having underlying conditions (72.4% vs 66.7%; *P* = .014) (Table 1). A larger percentage of NH-HCP than H-HCP reported working as a nursing assistant or patient care technician (38.9% vs 9.2%; *P* < .001).

### Close contact and practices in the workplace

NH-HCP were more likely than H-HCP to report close contact in the workplace with patients (50.5% vs 44.2%; *P* = .008) or coworkers or visitors (31.0% vs 14.8%; *P* < .001) with COVID-19 (Table 2). During care of patients with COVID-19, H-HCP were more likely than NH-HCP to report always using masks or respirators (94.3% vs 90.5%; *P* = .043), gowns (71.5% vs 61.3%; *P* = .002), or eye protection (70.4% vs 62.6%; *P* = .019) (Table 2). H-HCP were also more likely than NH-HCP to report having close contact with patients with COVID-19 who always had source control in place (21.4% vs 5.6%; *P* < .001).

**Table 2.** Close Contacts With Persons With COVID-19 and Practices and Activities In and Outside the Workplace Among Healthcare Personnel (HCP) With SARS-CoV-2 Infection^a^

Close Contact, Practice, or Activity	Hospital (N = 1,225)	Nursing Home (N = 604)	Other Facility (N = 789)	All Facilities (N = 2,625)	*P* Value^b^
**Close contacts with persons with COVID-19, no. (%)** ^c^					
In any setting	908 (74.1)	406 (67.2)	475 (60.2)	1,791 (68.2)	.004
In the workplace, with patients with COVID-19	541 (44.2)	305 (50.5)	164 (20.8)	1,010 (38.5)	.008
In the workplace, with someone with COVID-19 other than a patient^d^	82/554 (14.8)	44/142 (31.0)	41/241 (17.0)	167/937 (17.8)	<.001
Outside the workplace	349 (28.5)	51 (8.4)	270 (34.2)	670 (25.5)	…
With a family member with COVID-19	241 (19.7)	41 (6.8)	211 (26.7)	493 (18.8)	<.001
With someone with COVID-19 other than a family member	120 (9.8)	11 (1.8)	64 (8.1)	195 (7.4)	<.001
**In the workplace, no. (%)**					
Always used the following during care of patients with COVID-19:					
Mask or respirator	510/541 (94.3)	276/305 (90.5)	141/164 (86.0)	927/1,010 (91.8)	.043
Gloves	492/541 (90.9)	268/305 (87.9)	121/164 (73.8)	881/1,010 (87.2)	.140
Gown	387/541 (71.5)	187/305 (61.3)	52/164 (31.7)	626/1,010 (62.0)	.002
Goggles or face shield	381/541 (70.4)	191/305 (62.6)	52/164 (31.7)	624/1,010 (61.8)	.019
Always cared for patients with COVID-19 who had source control in place^e^	116/541 (21.4)	17/305 (5.6)	54/164 (32.9)	187/1,010 (18.5)	<.001
Always practiced social distancing with coworkers in the workplace	233 (19.0)	192 (31.8)	309 (39.2)	735 (28.0)	<.001
Had a mucous membrane or skin exposure to body fluids from patients with COVID-19	55/541 (10.2)	40/305 (13.1)	24/164 (14.6)	119/1,010 (11.8)	.023
Practiced extended use or reuse of a respirator during care of patients with COVID-19^f^	161/205 (78.5)	34/58 (58.6)	19/43 (44.2)	214/306 (69.9)	.002
Always practiced universal masking in the workplace	986 (80.5)	498 (82.5)	589 (74.7)	2,074 (79.0)	.325
**Outside the workplace, no. (%)**					
Attended a mass gathering or gathering with people other than household members	245 (20.0)	34 (5.6)	120 (15.2)	400 (15.2)	<.001
Traveled domestically or internationally	223 (18.2)	29 (4.8)	109 (13.8)	362 (13.8)	<.001
Used public or shared transportation	165 (13.5)	67 (11.1)	88 (11.2)	320 (12.2)	.323
Had close contact with ill person(s) outside of a healthcare facility	210 (17.1)	26 (4.3)	127 (16.1)	363 (13.8)	<.001

a With the exception of close contact in any setting, which could have been at any time before HCP interview, other close contacts, practices and activities occurred during the 14 days before or on the day of HCP SARS-CoV-2 infection onset, which was defined as the date of symptom onset or, in asymptomatic HCP, the date of specimen collection for the positive test.

b *P* value calculated with mid-*P* exact tests for the comparison of percentages of nursing home HCP responding yes or no/not sure/not reported versus hospital HCP responding yes or no/not sure/not reported.

c Close contact was defined as (1) being within 2 m of a person with COVID-19 for at least a few minutes (May–August 2020), for ≥15 min (August–November 2020), or for a total of ≥15 min in a 24-h period (after November 9, 2020); (2) having unprotected direct contact with infectious secretions or excretions; or (3) performance or participation in aerosol-generating procedures, regardless of the duration (starting in November 2020).

d Added to the HCP interview in August 2020; data were reported from a total of 554 hospital HCP, 142 nursing home HCP, and 241 other facility HCP.

e Patients with COVID-19 were considered having source control in place if they were wearing a face mask or cloth face covering or they were under invasive mechanical ventilation during close contact.

f Among HCP who reported using a respirator.

### Close contact and activities outside the workplace

Close contact with persons with COVID-19 and selected activities outside the workplace were more common among H-HCP than NH-HCP. A higher percentage of H-HCP than NH-HCP reported close contact with family members with COVID-19 (19.7% vs 6.8%; *P* < .001); attending mass gatherings or gatherings including people other than household members (20.0% vs 5.6%; *P* < .001); and traveling domestically or internationally (18.2% vs 4.3%; *P* < .001).

## Discussion

In this diverse cohort of HCP with SARS-CoV-2 infection, we observed that distribution of race and ethnicity and selected practices in and outside the workplace varied by healthcare setting. Approximately two-thirds of NH-HCP reported being part of racial and ethnic minority groups or having underlying conditions, which in some cases may be associated with an increased risk for infection or severe COVID-19.^4,5^ Culturally specific interventions and mitigation support among this HCP population should be considered.^6^

Our data confirm the workplace is an important source of SARS-CoV-2 infection among all HCP, and they suggest that it may be particularly important for NH-HCP, who were more likely to report close contact with patients or others with COVID-19 in the workplace and less likely to report always using recommended personal protective equipment (PPE). This finding is not surprising given the widely recognized challenges with testing, availability of infection prevention expertise and staffing, limited PPE supply, respiratory protection programs, and quarantine and isolation of residents in US nursing homes.^7^ To mitigate the effects of COVID-19 on NH-HCP and residents, the CDC and other partners have developed guidance and deployed resources and expertise to nursing homes around the country to bolster infection prevention and control.^8,9^

In contrast, exposures outside the workplace may be more common for H-HCP, who were more likely than NH-HCP to report close contact with family members or others with COVID-19 and participation in selected activities that may be associated with increased exposure risk. Focused messaging emphasizing community prevention practices may be needed.

Our findings have 2 main limitations. First, we analyzed data from a convenience sample of facilities and HCP; thus, results likely are not generalizable to all US HCP. Second, participation by HCP working in different healthcare settings was not proportionate across sites; therefore, differences observed between H-HCP and NH-HCP could be explained by other factors not accounted for in this analysis, such as differences among sites or healthcare settings in demographics, underlying conditions, or healthcare roles.

The US healthcare workforce is diverse, and protection of this workforce is essential to the safety and well-being of the nation. Recognition of the differences in workplace and community factors that affect the risk for SARS-CoV-2 infection among HCP may inform the development and delivery of effective public health outreach and messaging.
